# Molecular Interaction Studies of Trimethoxy Flavone with Human Serum Albumin

**DOI:** 10.1371/journal.pone.0008834

**Published:** 2010-01-21

**Authors:** Mahesh Gokara, Babu Sudhamalla, Damu G. Amooru, Rajagopal Subramanyam

**Affiliations:** 1 Department of Biochemistry, School of Life Sciences, University of Hyderabad, Hyderabad, India; 2 Department of Chemistry, Yogi Vemana University, Kadapa, India; Universita di Sassari, Italy

## Abstract

**Background:**

Human serum albumin (HSA) is the most abundant protein in blood plasma, having high affinity binding sites for several endogenous and exogenous compounds. Trimethoxy flavone (TMF) is a naturally occurring flavone isolated from *Andrographis viscosula* and used in the treatment of dyspepsia, influenza, malaria, respiratory functions and as an astringent and antidote for poisonous stings of some insects.

**Methodology/Principal Findings:**

The main aim of the experiment was to examine the interaction between TMF and HSA at physiological conditions. Upon addition of TMF to HSA, the fluorescence emission was quenched and the binding constant of TMF with HSA was found to be *K_TMF_* = 1.0±0.01×10^3^ M^−1^, which corresponds to −5.4 kcal M^−1^ of free energy. Micro-TOF Q mass spectrometry results showed a mass increase of from 66,513 Da (free HSA) to 66,823 Da (HAS +Drug), indicating the strong binding of TMF with HSA resulting in decrease of fluorescence. The HSA conformation was altered upon binding of TMF to HSA with decrease in α-helix and an increase in β-sheets and random coils suggesting partial unfolding of protein secondary structure. Molecular docking experiments found that TMF binds strongly with HSA at IIIA domain of hydrophobic pocket with hydrogen bond and hydrophobic interactions. Among which two hydrogen bonds are formed between O (19) of TMF to Arg 410, Tyr 411 and another one from O (7) of TMF to Asn 391, with bond distance of 2.1 Å, 3.6 Å and 2.6 Å, respectively.

**Conclusions/Significance:**

In view of the evidence presented, it is imperative to assign a greater role of HSA's as a carrier molecule for many drugs to understand the interactions of HSA with TMF will be pivotal in the design of new TMF-inspired drugs.

## Introduction

Flavonoids are naturally occurring polyphenolic compounds used as food supplements which has anti-allergic, anti-inflammatory, anti-microbial and anti-cancer activity. Dietary flavonoids can be detected in plasma as serum albumin-bound conjugates and are receiving increasing attention as potential prophylactics against a variety of human diseases, in particular cardiovascular disease and cancer [1]–[3]. A large number of mechanisms of action have been attributed to flavonoids, including antioxidant properties [4]–[7] and effects on enzymes and signal transduction pathways. The anti-oxidative protections are related to their binding modes to DNA duplex and complexation with free radicals *in vivo*. However, flavonoids are known to inhibit the activities of several enzymes such as calcium phospholipid-dependent protein kinase, tyrosine protein kinase from rat lung, phosphorylase kinase and DNA topoisomerases; these cases emphasize the additional importance of flavonoid-protein interactions [8]–[10].

Flavonoids display moderate affinities for albumins with binding constants in the range of 1–15×10^4^ M^−1^ [11]. Also, it is known that flavones and flavonols bind very tightly to albumin. Flavones are a class of flavonoids based on the backbone of 2-phenyl-1-benzopyran-4-one. Flavones which are used in anti-depression treatment are known to inhibit human cytochrome P450 (CYP) enzyme activities [12]. Hydrophobicity, the presence/absence of some functional groups, steric hindrance and spatial arrangement all play important roles in the affinity of natural polyphenols towards plasmatic proteins [13].

Tri-methoxy flavone (TMF) is a naturally-occurring compound present in *Andrographis viscosula*. This plant has been used in Ayurvedic medicine to cure many diseases. The plant's extract exhibits antityphoid and antifungal activities. This extract also reported to possess antihepatotoxic, anti-HIV, antibiotic, antimalarial, antihepatitic, antithrombogenic, antiinflammatory, anti-snake venom, and antipyretic properties to mention a few, besides its general use as an immuno-stimulant agent. Various species of *Andrographis* are used in the treatment of dyspepsia, influenza, malaria and respiratory functions and as a astringent and antidote for poisonous stings of some insects [14]–[16]. Although TMF has several clinical applications, however, its molecular interaction with human serum albumin has not been reported.

Human serum albumin is a major extracellular protein with high affinity for a wide range of metabolites and drugs [17]–[25] and its abundance is high in the blood plasma (40 mg ml^−1^) [26], [27]. The most important physiological roles of this protein are to bring such solutes into the bloodstream and deliver them to the target organs and to maintain the pH. In addition to its ordinary clinical applications such as hypovolemic shock treatment, many investigators have attempted to utilize HSA as carrier to deliver various drugs to their specific target organs. HSA is a single non-glycosylated, 67 kDa polypeptide, which folds into a heart shaped protein with approximately 67% α-helical content [28]–[34]. It is a globular protein composed of three structurally similar domains (I–III), each consisting of two subdomains (A and B) and stabilized by 17 disulfide bridges. The two primary binding sites (site I and site II) are hydrophobic cavities located in subdomains IIA and IIIA, respectively. Most compounds binds to these two sites with an affinity constant of 10^4^ to 10^6^ M^−1^ [28], [34], [35]. In addition, seven binding sites for fatty acids are localized in sub-domains IB, IIIA and IIIB, and on the sub-domain interfaces [27], [32], [36]. HSA also has a high-affinity metal binding site at the N-terminus [27]. There are many reports containing studies on HSA structure and its interactions with different ligands [18]–[24]. Very recent reports from our group [37], [38] show that the natural products of pentacyclic triterpenoids, betulinic acid and feruloyl maslinic acid isolated from *Tephrosia calophylla* and *Tetracera asiatica* form strongly-bound ligand–HSA complexes. Interactions with plasma proteins, especially HSA, are important factors to be considered in drug development. The interactions of TMF with HSA are significant for understanding its transport and distribution and to clarify its action mechanism and pharmaceutical dynamics.

In this study, the interactions of TMF with HSA was investigated by using fluorescence emission, circular dichroism (CD), micro-TOF Q mass spectrometry and molecular docking studies.

## Materials and Methods

### Isolation and Purification of TMF

**Figure 1:**
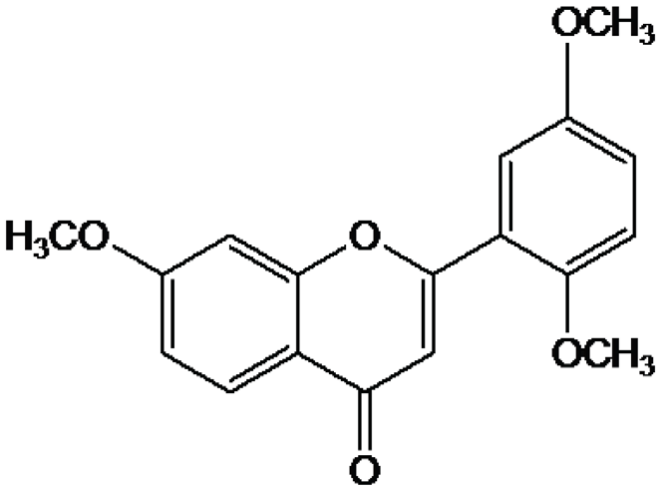
Chemical structure of Tri-methoxy flavone. The molecular mass is 312.31 Da and its molecular formula is C_18_H_16_O_5._

The whole plant of *A. viscosula* (2.5 Kg) was shade dried, powdered, and extracted with *n*-hexane (5133), Me_2_CO(5133), and MeOH(5133), successively. The *n*-hexane (30 g) was purified by column chromatography over silica gel, and eluted with a step gradient of *n*-hexane and EtOAc. The eluates at *n*-hexane∶EtOAc ratios of 8∶2, 7∶3, and 1∶1 were purified individually by repeated silica gel chromatography followed by preparative TLC (developed with benzene/EtOAc, 9∶1) to yield 10 mg of tri-methoxy flavones [16]. The ^13^C NMR spectrum shows that the structure of this fraction was established as 5, 7, 2′-trimethoxyflavone [16]. The molecular mass is 312.31 Da and its molecular formula is C_18_H_16_O_5_ (Figure 1).

### Preparation of Stock Solutions

Fat free human serum albumin (a kind gift from Virchow biotech Pvt Ltd, Hyderabad) was dissolved in physiological aqueous solution of 0.1 M phosphate buffer pH 7.2 to the final concentration of 1.5 mM protein according to the previous procedure [37]. It was observed that HSA is pure without any contamination. Fluorescent tri-methoxyflavone was prepared (2 mM) in 20∶80 ethanol: water mixture. From our previous work, a solution containing 20% ethanol has no effect [37], [38], on HSA secondary structure. The optimum physiological pH for HSA was set to 7.2 as it has the maximum absorption at this pH [37], thus, for all the experiments, we have used 0.1 M phosphate at pH 7.2 as a physiological buffer. The maximum binding time taken by the TMF to HSA was also examined via absorption, fluorescence, CD spectra, from the data it was found to be 10 min is the maximum binding time taken, hence we used 10 minutes incubation for all the experiments. All other chemicals are analytical grade purchased from Sigma Aldrich.

### Fluorescence Spectroscopy

The fluorescence emission spectra were recorded on a Jobin-Yvon, FluoroMax-3 in a 1 cm quartz cell using excitation wavelength of 285 nm. The excitation and emission slit widths are 5 nm and 5 nm, respectively. The emission spectra were recorded from 300 to 550 nm. The final concentrations of the TMF used for absorption spectra were 0.005, 0.01, 0.02, 0.03, 0.04, 0.05, 0.06, 0.07 and 0.08 mM in 10 mM phosphate buffer pH 7.2, and with a fixed HSA concentration 0.025 mM. TMF has fluorescence in the range of 451 nm. However the fluorescence of TMF has been subtracted from the HSA-TMF spectra.

### Electrospray Ionisation Mass Spectrometry (microTOF-Q)

Positive ion mode mass spectra were recorded on a micro-TOF Q (Bruker Daltonics, Bremen, Germany) equipped with an electrospray ionization source. For these measurements, the HSA concentration used was 0.15 µM and the TMF concentration was 0.2 µM. Free HSA and HSA–TMF were prepared in 5 mM ammonium acetate (pH 7.2) mixed with 20% acetonitrile, and introduced into the mass spectrometer source with a syringe pump (KD Scientifics Inc., Hilliston, MA) at 3 µL/min. Electrospray was performed by setting the spray voltage at 4.5 kV. The Time-of-Flight (TOF) pressure was maintained at less than 3×10^−7^ Torr. Scanning was performed over an *m*/*z* range of 50–3000, with collision energy of 10 eV. Data were averaged for 2 min and then smoothed using the Gaussian algorithm in the Bruker data analysis 3.4 software program. The instrument was calibrated using ES Tuning Mix (Agilent Technologies, part No. G2421-60001), diluted 1∶60 (v/v) times with 95% acetonitrile and injected through a divert valve just before sample application.

### Circular Dichorism Spectroscopy

Circular dichroism (CD) spectra of HSA and HSA–TMF were recorded with a Jasco J-810 spectropolarimeter, using a quartz cell with a path length of 0.02 cm. Five scans were accumulated at a scan speed of 50 nm min^−1^, with data being collected at every 1 nm from 190 to 300 nm. An ellipticity of CD spectra is expressed in millidegrees. The protein secondary structure was calculated using CDNN 2.1 software. For CD studies, the final concentration of HSA was 0.025 mM and spectra were recorded at TMF concentrations of 0.01, 0.025 and 0.08 mM. Temperatures of samples were maintained by Jasco J-715 peltier.

### Molecular Modeling and Docking

#### Genetic algorithm

GOLD (Genetic Optimization for Ligand Docking), a docking program based on genetic algorithm [39]–[42] was used to dock the ligands to the protein active sites. Genetic algorithm is a computer program that mimics the process of evolution by structures called chromosomes. Each of these encodes a possible solution (in terms of a possible ligand-receptor interaction) to the docking problem and may be assigned fitness score based on the relative merit of that solution. Each chromosome encodes an internal conformation and protein active site, and includes a mapping from hydrogen bonding sites in the ligand and protein. On decoding a chromosome, least-squares fitting process is employed to position the ligand within the active site of the protein. The fitness of a decoded chromosome is then a combination of the number and strength of the hydrogen bonds that have been formed in this way and of the vander Waals energy of the bound complex.

#### Preparation of the protein and the ligand

A crystal structure of HSA (PDB ID: 1AO6) was obtained from the Brookhaven Protein Data Bank. A three dimensional structure of TMF was built and the geometry optimized using the discover3 feature in the InsightII/Builder software package. Water molecules and ions were removed (including ordered water molecules) and hydrogen atoms added at appropriate geometry; groups within the protein were ionized as required at physiological pH. The structure of HSA was protonated in InsightII (www.accelrys.com). The genetic algorithm implemented in GOLDv3.2 was applied to calculate the possible binding conformations of the drug.

#### Genetic algorithm parameters used

The parameters used for genetic algorithm were active site radius-30; Population size-100; Number of Islands-5; Niche size-2; Selection pressure-1.1; Migrate-10; Number of operators-100,000; Mutate-95; Crossover-95. The default speed selection was used to avoid a potential reduction in docking accuracy. Fifty genetic algorithm runs with default parameter settings were performed without early termination. To estimate the protein-ligand complexes, scoring function, GOLD score was employed [41].

During docking process a maximum of 10 different conformations were considered for the TMF. Among which the best and the most three energetically favourable conformations with fairly similar GOLD fitness score of each ligand was selected. The conformer with the lowest binding free energy with highest fitness score was used for further analysis [41], [43].

## Results and Discussion

### Fluorescence Spectroscopy

Fluorescence emission spectroscopy was used to find the drug-protein interactions and measure binding affinity [44]. The emission fluorescence of HSA comes from tryptophan, tyrosine, and phenylalanine. Phenylalanine has a very low quantum yield and the fluorescence of tyrosine is almost totally quenched if it is ionized or present near to an amino group, a carboxyl group or a tryptophan. Thus, the fluorescence of HSA is dominated by the tryptophan emission, and the emission spectrum of HSA is mainly from a single residue Trp-214 in subdomain IIA. A change in the intrinsic fluorescence intensity of HSA was due to the tryptophan residue when small molecules bound to HSA [45].

**Figure 2:**
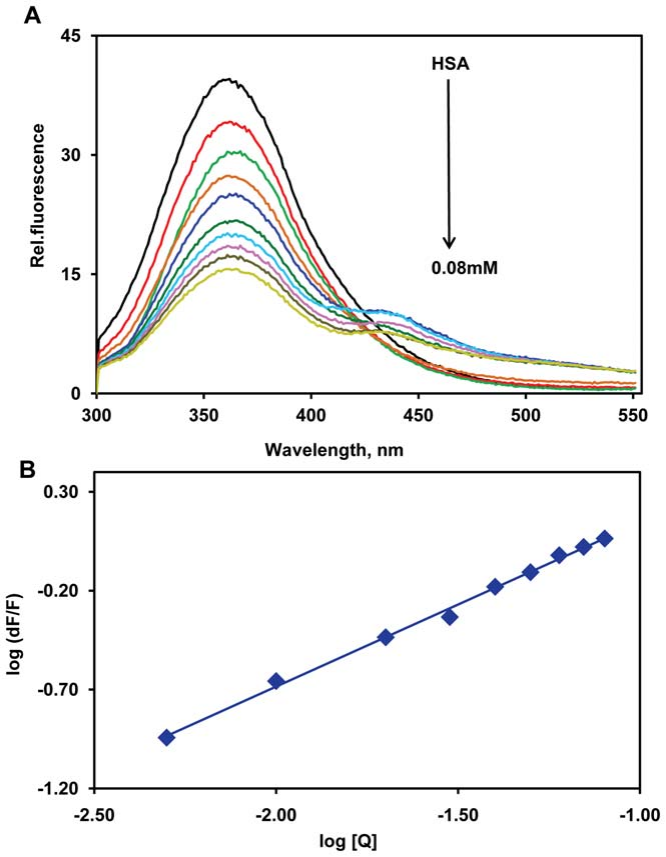
Fluorescence emission spectra of HSA–TMF in 0.1 M phosphate buffer pH 7.2, λ_ex_ = 285 nm, temperature = 25±1°C. A) Free HSA (0.025 mM) and free HSA with different concentrations of TMF (0.005, 0.01, 0.02, 0.03, 0.04, 0.05, 0.06, 0.07, 0.08 mM). B) Plot of log (dF/F) against log [Q]. λ_ex_ = 285 nm λ_em_ = 362 nm.

Figure. 2A shows the fluorescence emission of HSA is obtained at 362 nm. Different concentrations of TMF were used to study the interaction with HSA. Our results showed that, with increasing concentrations of TMF (0.005 to 0.08 mM) and a fixed concentration of HSA (0.025 mM), the maximum fluorescence (362 nm) of HSA was quenched upon binding of TMF (Figure. 2A). This indicates that TMF binding to HSA causes microenvironment changes in HSA and leads to HSA-TMF complexes. TMF also shows fluorescence emission at 451 nm (see Figure. 2A). Similar fluorescence quenching results were reported for several ligands [46], [47].

The binding constant can be calculated from the modified Stern-Volmer plot according to the following equation [48]

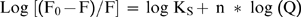
(1)where n is the slope (i.e. the number of binding sites), K_S_ is the binding constant and Q is the quencher concentration. The result indicated a good linear relationship (see Figure. 2B), suggesting that HSA interacts with TMF in a one-to-one ratio. The binding constant K*_TMF_* was calculated from the intercept as 1.0±0.01×10^3^ M^−1^, which indicates an adequate binding of TMF to the protein. The calculated binding constants show a comparatively weak ligand-protein interaction, corresponds to other strong ligand-protein complexes like monoclonal antibodies with binding constants from 10^7^ M^−1^ to 10^10^ M^−1^ [49]. It is important to note that natural products showed binding constants which are in the order of magnitude smaller than 10^7^ M^−1^ to 10^10^ M^−1^. Other flavonoids like, quercitin binds with an affinity of 1.46×10^4^ M^−1^ [50] and resveratrol a polyphenol binds with an affinity of 1.64×10^5^ M^−1^ [51].

The standard free energy change can be calculated according to:
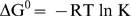
(2)where ΔG is the free energy, *K* is the binding constant at the corresponding temperature (which can be obtained from fluorescence data as described above) and *R* is the gas constant. Thus, the standard free energy change is calculated to be −5.4 kcal/mol at 25°C upon binding of TMF to HSA. This indicates that the free energy of binding for the TMF–HSA complex derives mainly from hydrophobic and possibly hydrogen bond interactions. Our data is in agreement with recently published values for the binding of silibinin and genistein to HSA [21], [52].

### Micro TOF-Q Analysis

**Figure 3:**
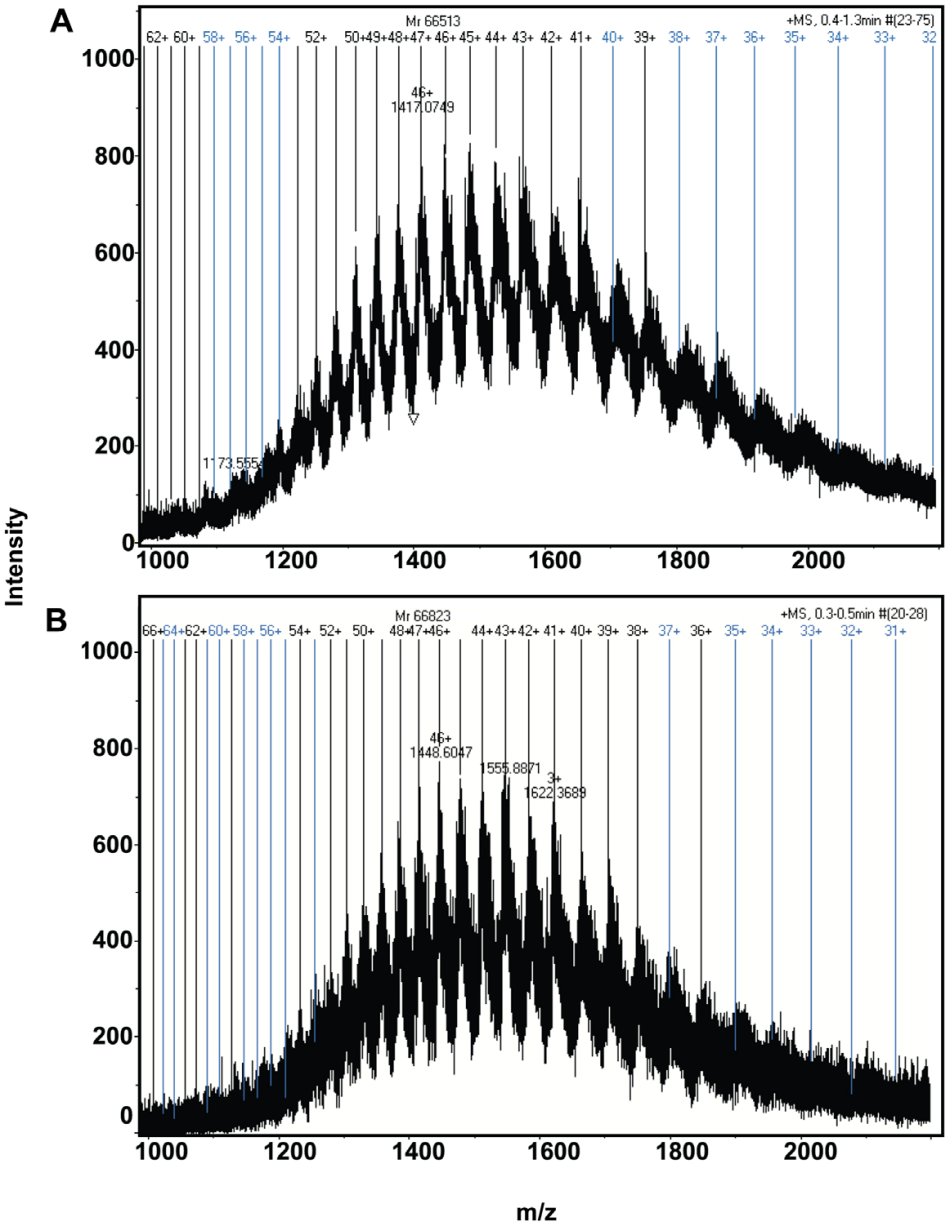
Micro TOF-Q mass spectra. A) free HSA, and B) HSA along with TMF. The concentration of free HSA and TMF were 0.15 µM and 0.2 µM, respectively.

Mass spectrometry is often used in pharmacokinetic studies due to its high sensitivity in detecting compounds at low concentrations. Protein–ligand complexation at micro molar levels was demonstrated here using micro TOF-Q mass spectrometry. The mass spectra of free HSA and HSA-TMF complexes can be observed in Figure 3A & 3B. The numbers on dark vertical lines indicate the matched charge states of HSA and HSA-TMF complexes. Deconvolution of the multiple charged states resulted in the mass determinations of HSA and HSA-TMF complexes. When analyzing the HSA-TMF sample a molecular mass increase from 66513 Da to 66823 Da was observed, indicating that TMF was bound to HSA. As the molecular weight of TMF is 312 Da the additional mass of 310 Da indicated that the interaction of TMF to HSA is 1∶1. These results are in agreement with the above fluorescence data. Our group recently illustrated the betulinic acid and feruloyl maslinic acid bound to the HSA in 1∶1 and 1∶2 showed by micro TOF-Q mass spectrometry [37], [38].

### CD Spectroscopy Studies

**Figure 4:**
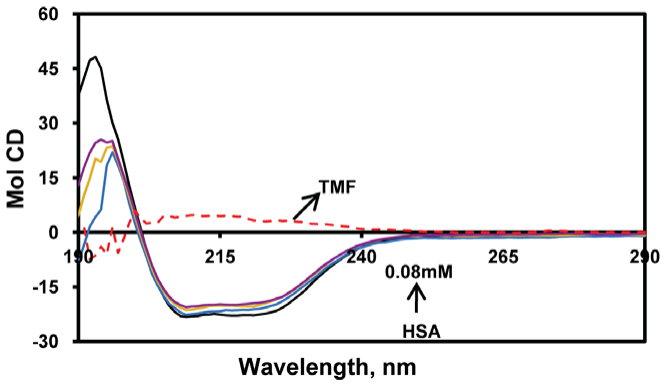
Circular dichroism of the free HSA and HSA+TMF complexes. The free HSA and HSA+TMF complexes in aqueous solution with a protein concentration of 0.025 mM and TMF concentrations were 0.01, 0.025 and 0.08 mM.

In general the CD spectroscopy used to study the secondary structure of proteins and their conformational changes. The CD spectra of HSA exhibits two negative bands in the ultraviolet region at 208 and 218 nm as shown in Figure 4. The secondary structure was determined using CDNN 2.1, software for protein secondary structure analysis. The secondary protein conformation found to be 57.3% α-helix, 24.9% β-sheet (including parallel and anti-parallel) and 17.8% random coil, respectively, which is in close agreement with our previous reports [37], [38]. By this method it was found that upon complexation of HSA with TMF (0.01, 0.025, 0.08 mM), the α-helical content of the protein decreased from 57.3% to 47% with a increase in β-sheets from 24.9% to 31.5%, and random coils 17.8% to 22%, respectively (**Table 1**). Our results suggest that the changes in the secondary structural components arise from partial unfolding of HSA upon binding of TMF. Several previous reports also indicate that conformational changes occur in HSA due to complexation with ligands [11], [36]–[38], [51], [53]–[55]. In our experiment, the near UV-CD shows that there is no modification in the tertiary structure upon complexation of TMF (data not shown). Thus, the protein conformation in our experiments arises due to change in the local structural changes in secondary structural components.

**Table 1:** Secondary structural analysis of the free HSA and its interaction with TMF.

	HSA	HSA-0.025 mM TMF	HSA-0.05 mM TMF	HSA-0.08 mM TMF
α–Helix (%)	57.3±2.5	51.1±2.25	48.1±2.0	47±2.0
Anti-parallel (%)	6.05±0.4	6.89±0.4	7.1±0.4	7.1±0.4
Parallel (%)	6.65±0.4	6.9±0.3	7.2±0.5	7.4±0.4
β–Sheet (%)	12.2±0.8	14.4±0.75	16.8±1.0	17±1.0
Random coil (%)	17.8±1.8	20.71±1.2	21.4±1.2	22±2.0

Based on the Figure 4, the data analyzed by web based software CDNN 2.1.

**Figure 5:**
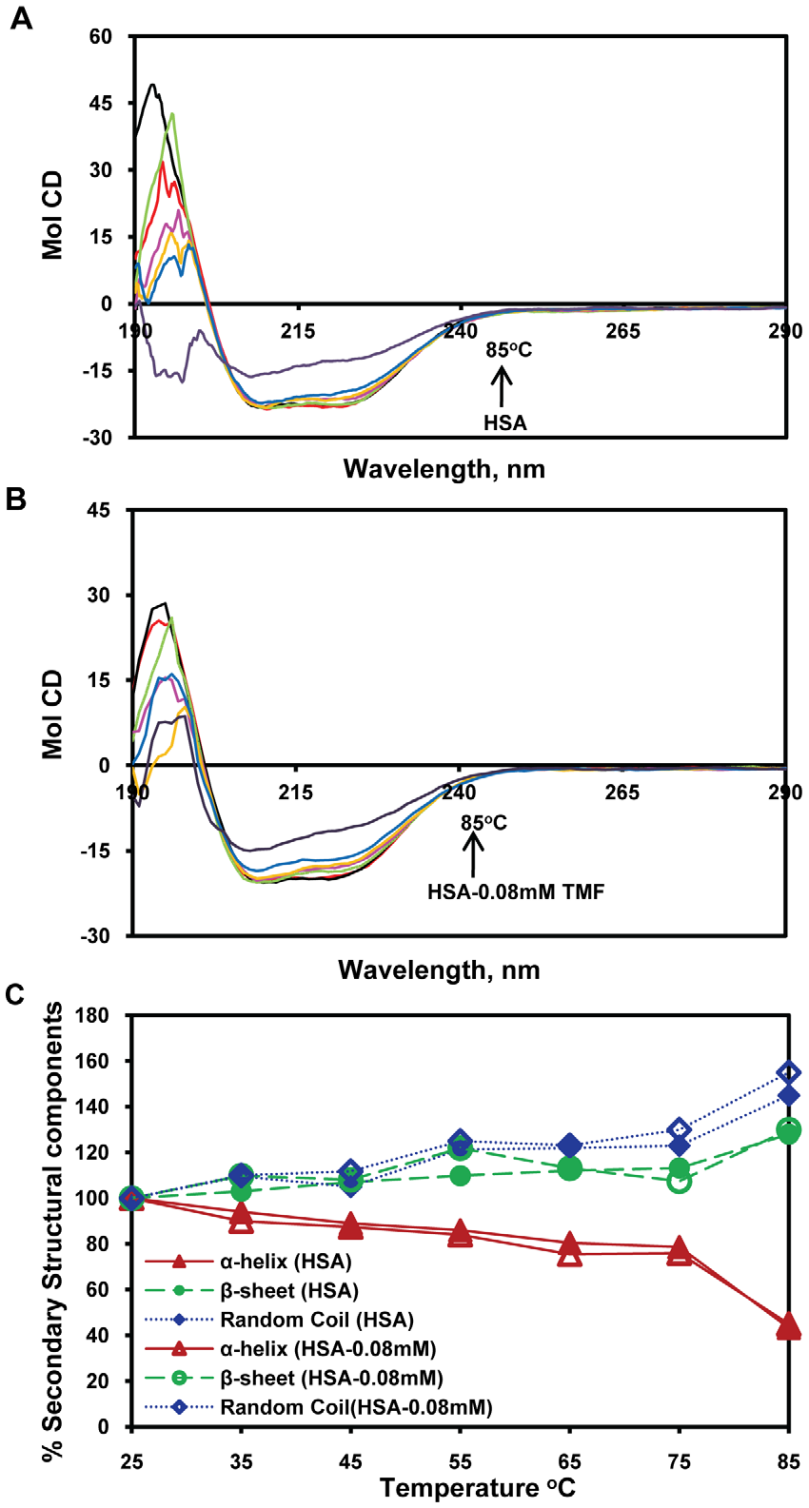
Temperature-dependent CD-Spectra of free HSA and HSA+TMF complexes. A) HSA alone and B) CD spectra of HSA+0.08 mM TMF complexes. C) Secondary structure composition calculated from Figure 5A, HSA alone and Figure 5B, HSA-TMF complexes with temperature dependent. The temperature dependance for both free HSA and HAS+TMF complexes from 25 to 85 with an interval of 10°C.

In order to determine the stability of HSA-TMF complexes, temperature-dependent CD was carried out for HSA alone (0.025 mM) (Figure 5A) and HSA with 0.08 mM TMF (Figure 5B), from 25–85°C. The secondary structural conformation of protein is not significantly changed up to 60°C in both free HSA alone and HAS+TMF complexes (Figure 5C). Above 65°C, the α-helical content decreased dramatically, while the β-sheet and random coil content increased in both HSA and HSA-TMF complexes. It is interesting to observe that HSA alone exhibits conformational changes from 65°C (Figure 5A). The previous report shows that the T_m_ of the HSA alone was around 65°C, which shows that the unfolding of protein occurs only after this point [56]. The secondary structural conformation was noticed in free HSA and TMF+HSA complexation (0.08 mM) that the α-helical contents were 57.3% and 47%, β-sheets 24.9% and 31.5%, and random coils 17.8% and 22%, respectively. The temperature dependent CD conformational changes remained same in both free HSA and HSA+TMF complexes, which indicate that there is no release of TMF from its complexation. These results indicate that the HSA-TMF complexes were not affected by temperature upto 60°C and thus, HSA-TMF complexes were conformationally and thermodynamically stable upto 60°C. Further, the protein conformation is dramatically decreased due to thermal denaturation.

### Molecular Docking

**Figure 6:**
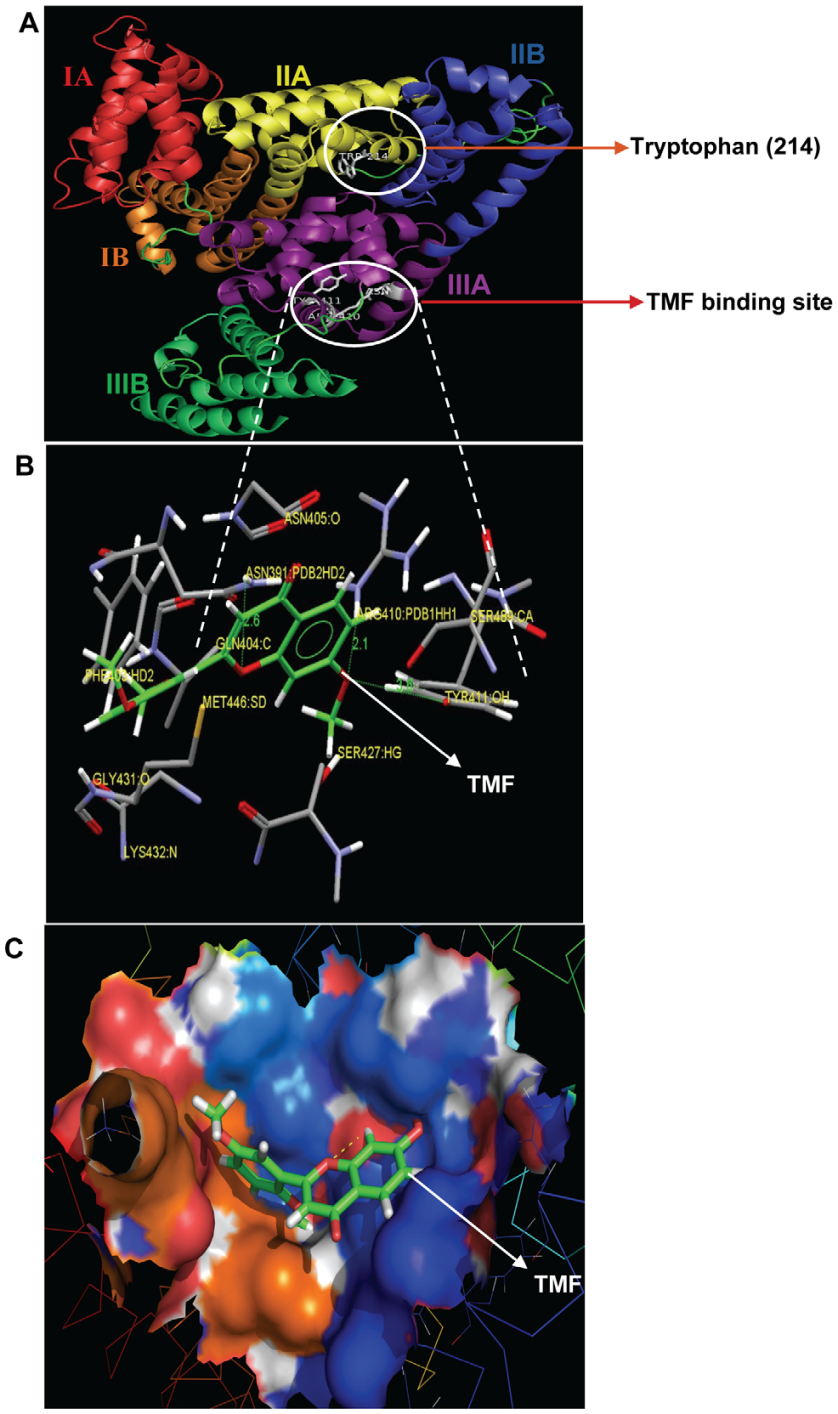
Molecular docking of HSA with TMF. A) Schematic representation of HSA molecule. Each subdomain is marked with a different colour (Red for subdomain IA; yellow, IIA; purple IIIA; blue, IIB; orange, IB; green, IIIB) Asn391, Arg410 and Tyr411 involved in Binding of TMF, Trp-214 are coloured white. B) Graphical representation of HSA-TMF complex (prepared by using SILVERv1.1.1 visualizer), TMF Complex represented as capped sticks, and the residues as ellipsoid model. Three H-bonds (as highlighted by the dashed lines in green colour) were formed between TMF and HSA. The hydrogen bond lengths were represented in green colour. C) Graphical representation of HSA showing TMF docked in the binding pocket of HSA using GOLDv3.2.TMF, depicted in stick model (light green), and HSA, represented in solid (better) with ray model. The image was visualised by using PyMol.

Computational molecular docking has been employed to improve the understanding of the interaction of TMF and HSA. As described above, the 3D crystal structure of HSA is a monomer consisting of three homologous domains which assemble to form a heart shaped molecule. Each of the structurally similar α-helical domains (I–III) has two subdomains (A and B), with six α-helices in subdomain A and four α-helices in subdomain B. The fluorescent tryptophan residue 214 is in subdomain IIA [28]. Several studies have shown that HSA is able to bind many ligands in several binding sites [34]. In the present study, the GOLD v3.2, was chosen to examine the binding mode of TMF at active site of HSA. The figure 6 shows the location of tryptophan and also the binding of TMF (Figure 6A & 6B).

The outer surface of the IIA and IIIA subdomains exhibits several hydrophobic pockets [20] and most ligands bind to this region. The present study indicates that TMF binds within the hydrophobic pocket of subdomain IIIA (see Figure 6C). The side chain of Arg410 is located at the mouth of the pocket while the hydroxyl group of Tyr411 faces toward the inside of the pocket. The complexation of HSA-TMF is stabilised primarily by three hydrogen-bond interactions. The hydrogen bonds are formed with the O(7) of TMF and Asn391 with a hydrogen bond length of 2.6 Å, another two H-bonds between O(19) of TMF and Arg410, Tyr411 with bond distances of 2.1 Å, 3.6 Å respectively (see Figure 6B & 6C). The fluorescence quenching occurred due to Trp-214 in IIA domain, that any perturbation in Trp-214 in subdomain IIA may induce changes in IIIA domain as well [57]. The results suggest that the formation of new hydrogen bonds decreased the overall hydrophilicity and increased the hydrophobicity, stabilizing the TMF–HSA complexes [21], [52]. The computationally calculated free energy change Δ*G* ^0^ for TMF-HSA binding is −6.1 kcal.mol^−1^, and the binding constant is K_T*MF*_ 1.2×10^3^ M^−1^. These results are very close to the experimentally-measured values of Δ*G* ^0^ = −5.4 kcal/mol and K_T*MF*_ = 1.0±0.01×10^3^ M^−1^. Therefore, the molecular docking and free energy calculation results suggested that TMF bound to HSA with both hydrophobic and hydrogen bond interactions.

In conclusion, TMF from *Andrographis viscosula* Nees binds to HSA with an affinity of K_T*MF*_ = 1.0±0.01×10^3^ M^−1^ and a binding free energy of −5.4 kcal M^−1^ at 25°C. Circular dichroism results indicate partial unfolding of the protein upon TMF binding and also indicated that these complexes are conformationally and thermodynamically stable. Mass spectrometry data reveals the additional mass increase is due to 1∶1 interaction of HSA to TMF. Further, molecular docking studies concluded that TMF-HSA complex is stabilised by three hydrogen bonds: one between the O(7) of the TMF and Asn391 with a bond length of 2.6 Å and two H-bonds between O(19) of TMF and Arg410 and Tyr411 with bond distances of 2.1 Å and 3.6 Å respectively. Nonetheless, the TMF was bound to HSA mainly by hydrogen and hydrophobic interactions. The biological importance of this study lies in HSA's role as a carrier molecule for many drugs–understanding the interactions of HSA with TMF will be essential in the design of new TMF-inspired drugs. This approach to drug development based on natural products and traditional medicine could be a major development in the pharmaceutical industry, chemistry, life sciences and clinical medicine.
